# Risperidone long-acting injection in Schizophrenia Spectrum Illnesses compared to first generation depot antipsychotics in an outpatient setting in Canada

**DOI:** 10.1186/1471-244X-13-155

**Published:** 2013-05-30

**Authors:** Laura Lammers, Bree Zehm, Richard Williams

**Affiliations:** 1 Pharmacy Department, Nanaimo Regional General Hospital, Vancouver Island Health Authority, Nanaimo, Canada; 2 Pharmacy Department, Royal Jubilee Hospital, Vancouver Island Health Authority, Victoria, Canada; 3 Department of Psychiatry, Eric Martin Pavilion, Vancouver Island Health Authority, Victoria, Canada

**Keywords:** Risperidone long acting injection, Atypical depot antipsychotic, First generation depot antipsychotic, Typical depot antipsychotic, Hospitalization, Treatment discontinuation, Retrospective

## Abstract

**Background:**

Depot formulations of antipsychotics provide a potential solution to the poor adherence to oral therapies in schizophrenia. However, there have been few comparative studies on the effectiveness and tolerability of first and second generation depot antipsychotics in a real clinical practice setting. The objectives of the present study were to compare safety and outcomes in patients with schizophrenia initiated on risperidone long-acting injection (RLAI) or first generation antipsychotic injections (FGAI) at a Mental Health Centre in British Columbia.

**Methods:**

Data were collected by retrospective chart review of all active patients starting depot therapy who were ≥ 18 years of age, had received at least 3 injections of depot antipsychotic and had no prior clozapine treatment. Kaplan Meier survival curves were used to estimate probability of treatment discontinuation and hospitalization.

**Results:**

A total of 70 RLAI and 102 FGAI patient charts were reviewed. At baseline patients in both groups had similar ages (39.7 and 42.7 years for RLAI and FGAI patients (p = 0.09), respectively) but FGAI patients had a longer time since diagnosis (13.6 vs. 9.85 years (p = 0.003)). Treatment retention at 18 months was 77% for RLAI and 86% for FGAI patients (p = 0.22) and 82% and 88% of patients, respectively (p = 0.28), had not been hospitalized. However, RLAI analyses were compromised by lack of long-term patient data. Concomitant medication utilization was similar in both groups except for anticholinergics which were used less frequently in RLAI patients (5.7% vs. 35.3%, p < 0.001). Adverse event frequency was also similar except for extrapyramidal symptoms (EPS) which were more common in FGAI patients (52.9% vs. 17.0% for RLAI (p < 0.001)).

**Conclusions:**

There was no apparent difference in treatment discontinuation or hospitalization between RLAI and FGAI treated patients, although analysis was compromised by low patient numbers. However, decreased EPS with RLAI may offer a significant clinical benefit to patients with schizophrenia.

## Background

Schizophrenia is a chronic mental illness with a range of symptoms which can lead to severe impairments in personal, social and occupational functioning [1]. The lifetime prevalence of schizophrenia is estimated at 0.55% [2] which correlates to approximately 185,000 Canadians with the disease. The disorder usually presents in early adulthood and as many as 50-70% of patients will suffer chronic relapses which lead to increased rates of hospitalization and extended morbidity and mortality [2,3]. The resulting economic cost is substantial, primarily resulting from the extensive hospitalization of patients during relapse (approximately 50% of the cost), while medication contributes approximately 6% of the overall cost [4].

One of the contributing factors to relapse in schizophrenia is poor or partial adherence to medication [5,6]. Although adherence in schizophrenia is a complex phenomenon, route of administration (oral vs. depot), treatment related side-effects and the effectiveness of medication are key factors in defining levels of long-term adherence [5,7,8]. Depot antipsychotics are often initiated in patients who have shown evidence of poor adherence or tolerability to oral medications [5]. However, although depot formulations of first generation or typical antipsychotics may have a positive impact on adherence, like their oral counterparts they have minimal impact on negative symptoms of schizophrenia and cognition and are associated with an increased risk of extrapyramidal symptoms (EPS) and tardive dyskinesia (TD) [9,10]. These limitations reduce the capacity of both oral and depot formulations of typical antipsychotics to provide effective control of schizophrenia [10].

Second generation or atypical antipsychotics are effective in the control of both positive and negative symptoms of schizophrenia and generally have a lower incidence of EPS and TD and in some patients can provide clinical advantages over typical agents [9,11-13]. Risperidone long-acting injection (RLAI) was the first atypical antipsychotic available in a depot formulation and the efficacy and tolerability of this agent have been demonstrated in clinical trials [14,15]. However, there is a lack of information on the comparative effectiveness and tolerability of typical and atypical depot formulations of antipsychotics when used in a real world clinical practice setting. In the present study, retrospective chart review was used to assess the impact of RLAI and first generation antipsychotic depot injections (FGAI) on patient outcomes at a Mental Health Centre in Canada.

## Methods

A retrospective chart review was conducted from December 2008 to March 2009 on all active patients at the Victoria Mental Health Centre, Partnership Medication Clinic (PMC), Victoria, British Columbia. The PMC is a primary location for the administration of depot antipsychotic medications to outpatients. The primary outcomes of this retrospective chart review were time to treatment discontinuation and time to hospitalization for patients on RLAI versus FGAI. Secondary outcomes included assessment of adverse side effects of therapy, including EPS and TD, duration of hospitalization and use of concomitant medications.

The inclusion criteria were patients starting depot therapy who were ≥ 18 years of age, having a DSM IV diagnosis of schizophrenia, schizophreniform disorder, or schizoaffective disorder, who had at least 3 injections of a depot antipsychotic. Exclusion criteria included evidence of a prior clozapine trial, documentation of active alcohol or substance abuse, or current pregnancy/lactation. Charts were examined based on a complete list of active patients at the PMC. The goal was to select all patients on RLAI and match them with an equal number of patients on an FGAI. However, because of the relatively small number of patients available, all patients at the PMC meeting the inclusion criteria were included. Charts were examined for the primary and secondary outcomes for the first 18 months of exposure to FGAI and/or RLAI.

Data collected included current antipsychotic medication and dose, concomitant psychiatric medications, patient age, gender, weight, time since diagnosis and comorbidities. For patients who had exposure to both RLAI and FGAI throughout their treatment history, information was recorded for each depot type, such that patients could be included in both groups. Among patients who were exposed to multiple FGAIs, only the first medication encounter was recorded. During the exposure time to antipsychotic, the most recent Abnormal Involuntary Movement Scale (AIMS) [16] and Simpson Angus Scale (SAS) [17] scores were recorded, if available from the chart. Cases of TD were identified if the score on the AIMS met the Schooler Kane criteria (moderate dyskinetic movement in one body area or mild dyskinetic movement in two body areas) [18] or the patient was reported to have TD. Patients were considered to have EPS if they were using an anticholinergic medication or met the definition for EPS (mean global score of 0.3 or more on the SAS, or a raw score of three) [17]. Time to treatment discontinuation was assessed over the entire period a patient had exposure to antipsychotic, regardless of a hospitalization. If after hospitalization the patient remained on the same depot antipsychotic, they were considered survivors for the discontinuation group, as depot therapy with the same agent continued. Patients who discontinued depot antipsychotic by choice and then relapsed or were hospitalized after a period of time were counted as a treatment discontinuation. All collected data were entered into a Microsoft Access Database.

Kaplan Meier survival curves were used to estimate the probability of treatment discontinuation and hospitalization at 18 months. In addition, Cox proportional hazard modelling was conducted to identify patient baseline characteristics that were significant predictors of treatment discontinuation or hospitalization. Comparisons between treatment groups for categorical variables were analyzed by the Pearson chi-square or Fisher’s exact method. For continuous variables with a parametric distribution, the student’s t-test was used; for nonparametric variables, the Kolmogorov-Smirnov (KS) test was used. P values below 0.05 were considered statistically significant; however, due to the preliminary nature of this study, trends in the data were also considered. Information was analyzed using SPSS Statistical Software (version 17.0) and all statistical tests were two-sided. Power was not calculated *a priori* as this was a hypothesis generating preliminary study.

Ethics approval for the study was granted by the Health Research Ethics Board of the Vancouver Island Health Authority. Informed consent was not required for this retrospective chart review.

## Results

**Figure 1:**
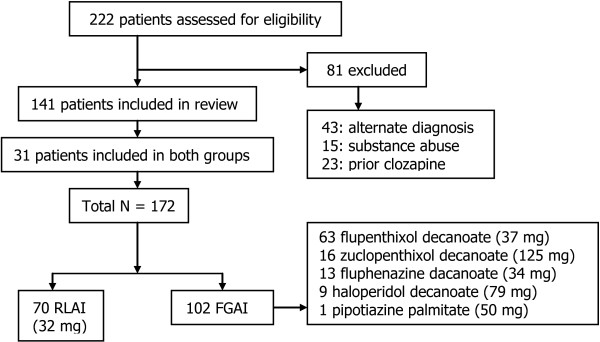
**Summary of chart review process.** Mean dose per injection for each depot antipsychotic is shown in parenthesis; frequency of administration was as per the respective Product Monographs.

As shown in Figure 1, 222 patient charts were eligible for review but 81 were excluded because they did not meet study inclusion criteria. Of the remaining patients (n = 141), 70 received RLAI and 102 FGAI while 31 patients were included in both treatment groups. At baseline, patients in both treatment groups had similar characteristics except for time since diagnosis where the FGAI patients had a significantly longer duration of diagnosis (13.6 years vs. 9.85 years for the RLAI group, p = 0.003) (Table 1). Duration of follow-up was 15.9 ± 4.6 months for FGAI and 11.2 ± 5.6 months for RLAI. The most common FGAI utilized was flupenthixol decanoate (63 of FGAI patients (61.8%) at a mean dose of 37 mg) while only 9 patients (8.8%) received haloperidol decanoate at a mean dose of 79 mg, the mean RLAI dose was 32 mg (Figure 1). See Table 2 for doses of depot, oral and total antipsychotics in CPZ equivalents.

**Table 1:** Baseline characteristics of patients

**Characteristic**	RLAI* (n = 70)	**FGAI (n = 102)**	**P value**
Age (Years)	39.7	42.7	0.09
Gender (n (%) male)	45 (64.3)	63 (61.8)	0.74
Weight (Kg)	90.61^†^	89.88^†^	0.97
Time since diagnosis (Years)	9.85	13.6	0.003

*Abbreviations are; *RLAI* Risperidone long-acting injection, *FGAI* First generation antipsychotic injection.

^†^Weight based on data on 41 and 52 patients for RLAI and FGAI, respectively.

**Table 2:** Does of antipsychotics for RLAI* and FGAI in CPZ equivalents [19]

	**RLAI Mean ± SD (mg)**	**FGAI Mean ± SD (mg)**
Depot Antipsychotics	386 ± 113	535 ± 368
*Flupenthixol decanoate*		*550 ± 368*
*Zuclopenthixol decanoate*		*376 ± 193*
*Fluphenazine decanoate*		*824 ± 572*
*Haloperidol decanoate*		*316 ± 120*
*Pipotiazine palmitate*		*300 ± 0*
*Risperidone microspheres*	*386 ± 113*	
Oral antipsychotics	316 ± 220	370 ± 335
Depot + oral antipsychotics	505 ± 283	716 ± 596

* Abbreviations are: *RLAI* Risperidone long-acting injection, *FGAI* First generation antipsychotic injection, *CPZ* chlorpromazine.

**Figure 2:**
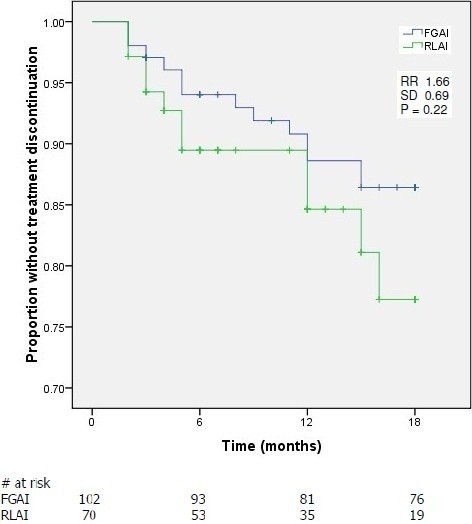
**Kaplan Meier survival curve for time to treatment discontinuation.** Survival curves are shown for RLAI (risperidone long-acting injection) and FGAI (first generation antipsychotic injection) treated patients. Abbreviations are; RR, Risk Ratio; SD, standard deviation.

The Kaplan Meier survival curves for treatment discontinuation were similar for both groups with 77% of RLAI and 86% of FGAI patients continuing treatment at 18 months (p = 0.22) (Figure 2). The mean survival time for RLAI and FGAI was 16.0 (95% confidence interval (CI), 14.9 to 17.1) and 16.7 (95% CI, 15.9 to 17.4) months, respectively, based on the Kaplan Meier analysis. However, few RLAI patients had long-term drug exposure; at 18-months only 19 patients were at risk in the Kaplan Meier analysis vs. 76 for FGAI (Figure 2). Further information on those who discontinued therapy is provided in Table 3 and indicates no significant differences in proportion discontinued, the time to discontinuation or in the major reasons for discontinuation between the FGAI and RLAI treatment groups. Although there were differences in the reasons for discontinuation between groups relating to patient choice (63.6% of RLAI discontinuations vs. 38.5% for FGAI) and tolerability (18.2% vs. 30.8%), none reached statistical significance.

**Table 3:** Medication discontinuation, hospitalization patterns and concomitant medication utilization in RLAI* and FGAI treatment groups

**Outcome**	**RLAI (n = 70)**	**FGAI (n = 102)**	**P value**
**Discontinuation**			
Proportion of patients discontinued (n (%))	11 (15.7)	13 (12.7)	0.30
Mean time to discontinuation (Months ± SD)	7.2 ± 5.4	7.9 ± 4.8	0.99
Reason for discontinuation (n (%))			
Patient choice	7 (63.6)	5 (38.5)	0.22
Intolerability	2 (18.2)	4 (30.8)	0.65
Treatment failure	2 (18.2)	0 (0)	0.20
Unknown	0 (0)	4 (30.8)	0.49
**Hospitalization**			
Proportion of patients hospitalized (n (%))	10 (14.3)	13 (12.7)	0.09
Mean time to hospitalization (Months ± SD)	5.4 ± 2.8	5.8 ± 5.1	0.80
Mean duration of hospitalization (Days ± SD)	35 ± 24	29 ± 17	0.49
Reason for hospitalization (n (%))			
Treatment failure	10 (100)	13 (100)	1.00
**Concomitant medications (n (%))**			
Additional antipsychotic	21 (30)	42 (41.1)	0.14
Anticholinergic	4 (5.7)	36 (35.3)	<0.001
Mood stabilizer	13 (18.6)	11 (10.8)	0.15
Antidepressant	10 (14.3)	26 (25.5)	0.08
Benzodiazepine	7 (10.0)	9 (8.8)	0.79

*Abbreviations are; *RLAI* Risperidone long-acting injection, *FGAI* First generation antipsychotic injection, *SD* standard deviation.

**Figure 3:**
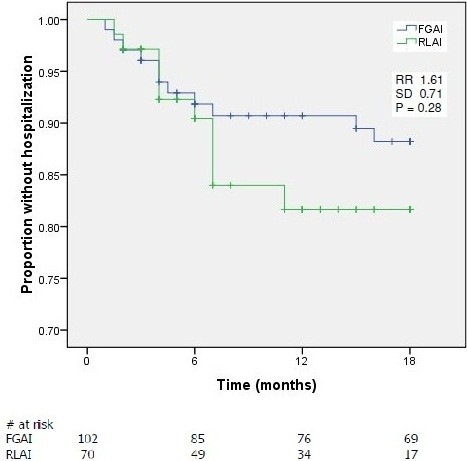
**Kaplan Meier survival curve for time to hospitalization.** Survival curves are shown for RLAI (risperidone long-acting injection) and FGAI (first generation antipsychotic injection) treated patients. Abbreviations are; RR, Risk Ratio; SD, standard deviation.

Kaplan Meier analysis of time to hospitalization was similar for both groups with 82% and 88% of patients in the RLAI and FGAI groups, respectively, without a relapse requiring hospitalization over the 18 month assessment period (p = 0.28) (Figure 3). The mean survival time for hospitalization was 15.8 (95% CI, 14.5 to 17.0) and 16.6 (95% CI, 15.8 to 17.4) months for RLAI and FGAI patients, respectively. For those patients who were hospitalized there were no significant differences in time to first hospitalization or in mean duration of hospitalization between RLAI and FGAI treatment (Table 3). In both treatment groups the reason for hospitalization was treatment failure. Cox proportional hazards models indicated that no assessed baseline characteristic (age, gender, time since diagnosis, patient weight or treatment with RLAI vs. FGAI) was a significant predictor of either treatment discontinuation or hospitalization (results not shown).

During treatment with both depot formulations there was extensive additional use of oral antipsychotics and psychiatric medications (Table 3). In the FGAI group, there was greater utilization of additional oral antipsychotics (41.1% of FGAI patients vs. 30% of RLAI patients), antidepressants (25.5% vs. 14.3%) and anticholinergics (35.3% vs. 5.7%), but only the latter was statistically significant (p < 0.001) with a 6.2-fold increase in anticholinergic use in FGAI patients. The most commonly used adjunct oral antipsychotics were quetiapine and olanzapine while procyclidine and benztropine were the most common anticholinergics. However, additional utilization of oral antipsychotics had no impact on the outcomes assessed. A sub-group analysis of the 70% and 59% of RLAI and FGAI treatment groups, respectively, who were receiving mono depot therapy (i.e., no additional oral antipsychotics) produced similar results for time to discontinuation and hospitalization to those already described for the overall analyses (results not shown).

As shown in Table 4, chart review indicated that similar proportions of both RLAI and FGAI patients had AIMS and SAS assessments. Both mean AIMS and SAS scores were higher for the FGAI treatment group, but the differences were not statistically significant. Nevertheless, the SAS assessment indicated a 3.1-fold increase (p < 0.001) in the incidence of EPS in the FGAI treated group (52.9% vs. 17% of RLAI patients). Also, more patients in the FGAI group had TD (4.9% vs. 1.4% in RLAI patients) but this difference was not statistically significant (p = 0.40). Results were similar for patients in both treatment groups receiving RLAI or FGAI monotherapy without additional oral antipsychotics (results not shown).

**Table 4:** Abnormal movement assessments and side effects of RLAI* and FGAI therapy

**Treatment outcome**	**RLAI**	**FGAI**	**P value**
**Abnormal movement**			
AIMS score completed (n (%))	36 (51.4)	61 (59.8)	0.28
Mean AIMS score	1.83	3.25	0.25
Proportion of patients with TD (n (%))	1 (1.4)	5 (4.9)	0.40
SAS score completed	33 (47.1)	54 (52.9)	0.45
Mean SAS score	2.82	4.85	0.28
Proportion of patients with EPS	12 (17.0)	54 (52.9)	<0.001
**Side effects of therapy (n (%))**			
Patients reporting side effects	7 (10.0)	17 (16.7)	0.21
EPS	1 (14.3)	3 (17.6)	
Akathesia	1 (14.3)	2 (11.8)	
Weight gain	2 (28.6)	2 (11.8)	
Tremor	0 (0)	4 (23.5)	
Galactorrhea	1 (14.3)	0 (0)	
Other^†^	2 (28.6)	6 (35.5)	

* Abbreviations are: *RLAI* Risperidone long-acting injection, *FGAI* First generation antipsychotic injection, *AIMS* Abnormal Involuntary Movement Scale, *TD* tardive dyskinesia, *SAS* Simpson Angus Scale, *EPS* extrapyramidal symptoms.

^†^Other includes injection site pain, dizziness, fatigue, TD (FGAI) and mental impairment (RLAI).

Reports of side effects of therapy recorded in patient charts confirmed a higher incidence of movement disorders in FGAI treated patients with 17.6% (vs. 14.3% for RLAI) and 23.5% (vs. 0% for RLAI) of patients with EPS and tremor, respectively (Table 4). There were no major differences in additional reported side effects between treatment groups, although only 10% and 16.7% of RLAI and FGAI patients, respectively, had such events recorded in their charts.

## Discussion

The aim of this study was to assess the impact of typical and atypical depot antipsychotics on schizophrenia patient outcomes in a real world clinical practice setting over an 18 month period. Retrospective chart review showed high levels of continuation on depot therapy with both FGAI and RLAI (86% and 77%, respectively, p = 0.22) and relatively low rates of hospitalization (12.7% and 14.3%, respectively, p = 0.09) with no significant differences between the two formulations. However, with RLAI there was a significantly lower incidence of EPS (17% vs. 52.9%, p < 0.001) and evidence suggesting reduced rates of TD and a lower utilization of oral antipsychotics, although these differences were not statistically significant.

Few studies have compared treatment retention and hospitalization with FGAIs and RLAI in similar patient populations in this type of clinical setting. However, observational studies have reported variable treatment retention rates; ranging from 49.8% over 36 months for depot typical [20] and up to 85% for RLAI [21] over 24 months, although estimation of such rates in schizophrenia are prone to differences in clinical practice patterns and disease severity in treated patients [21]. In addition, numerous studies have demonstrated that a switch from oral antipsychotics to typical or atypical depot formulations can lead to decreases in patient hospitalization rates [21,22]. However, using a similar retrospective chart audit methodology, Virit et al. [23] assessed outcomes in schizophrenia patients in Turkey who were switched from oral antipsychotics to FGAIs (n = 46) or RLAI (n = 22). After mean treatment periods of 20.4 and 15.2 months for FGAIs and RLAI (p = 0.02), respectively, 90.9% of the latter patients were still on therapy compared to 58.7% for FGAI (p = 0.02). This suggested improved retention with RLAI, although results may have been compromised by the lack of long-term treatment data for RLAI because of its relatively recent availability compared to FGAIs [23]. A similar problem occurred in the present study where fewer RLAI patients had long-term exposure to therapy compared to FGAIs; only 17% of RLAI patients had exposure beyond 18-months compared to 72% of FGAI patients and average exposure times were 12 and 64 months, respectively. As a result the current analysis was restricted to a comparison over 18-months so that groups could be compared on similar terms. The limited number of patients with long-term RLAI data may have impacted the validity of treatment retention and hospitalization estimates in this population. Virit et al. [23] suggest that treatment retention on RLAI in their study may be higher than with FGAIs because of the significantly lower incidence of adverse events (p = 0.02) with the former antipsychotic.

In contrast, using a different methodology, Olfson et al. [24] found no difference in treatment retention rates between FGAIs and RLAI. By analysis of an administrative Medicaid database in the United States the authors reported that 6 months after treatment initiation, only 9.7%, 5.4% and 2.6% of patients were continuing on haloperidol decanoate, fluphenazine decanoate and RLAI, respectively, rates much lower than those reported here. However, apart from the recognised limitations of analyzing administrative databases [24], the patients in this study also had high levels of polypharmacy (≥ 70% to 93% of patients also received oral antipsychotics during depot therapy), suggesting extensive psychiatric comorbidity which may have had a negative impact on treatment retention [24].

In the present study 30% and 41.1% of RLAI and FGAI patients, respectively, were using additional oral antipsychotics. In clinical practice, antipsychotic polypharmacy has been reported to range from 13% to 60% and can have a significant impact on schizophrenia patient outcomes [25,26]. However, in the present study polypharmacy did not appear to impact the observed outcomes. When the analyses were repeated on patients receiving RLAI or FGAI monotherapy, all outcomes, including incidence of movement disorders, were unchanged suggesting that oral antipsychotics played no role in defining treatment retention, hospitalization or adverse events. In fact, the majority of additional antipsychotic prescriptions were for quetiapine dosed at bedtime, indicating likely use as a sleep aid.

Both the RLAI and FGAI patient groups had similar characteristics at baseline, although time since diagnosis was significantly longer in the latter group (3.75 years longer). Therefore, it is possible that the FGAI group were at a more stable course in their disease and hence less prone to relapses, compared to the RLAI group, who with a more recent diagnosis may have had more unstable disease. Similarly, although not statistically significant, the RLAI group was on average three years younger, again suggesting a possible difference in disease course between the two groups. These differences may have contributed to the observed RLAI and FGAI outcomes.

The present study presents clear evidence that treatment with RLAI in an outpatient setting leads to significantly lower rates of EPS and a trend towards a lower rate of TD, compared to conventional depot antipsychotics. This confirms previous studies which have shown significant reductions in EPS and movement disorders following a switch from FGAIs to RLAI [27,28]. Although not assessed in the present study, switching from a typical depot to RLAI has also been shown to result in significant improvement in Positive and Negative Syndrome Scale (PANSS) scores suggesting a broad impact of RLAI on symptom control in schizophrenia [27,28]. In the present study, incidence of movement disorders was based mainly on AIMS and SAS assessments recorded in patient charts. However, only 56% and 50% of patients had AIMS and SAS evaluations, respectively, despite Canadian Psychiatric Association guidelines which recommend assessment every 6 months [29]. This suggests that these recommendations are not universally applied in clinical practice. Symptoms of EPS have a large impact on patient functioning and can also affect adherence to antipsychotic medication, hence the recommendation for frequent assessment [29-31]. In the present study, the observation that RLAI significantly decreases the incidence of EPS compared to FGAIs, while providing high levels of symptom control, may have significant implications for long-term patient care with depot antipsychotics.

There are a number of limitations associated with this study. Firstly, data were only collected from patients at the PMC currently receiving depot injections. As a result, patients who tried depots and relapsed, or discontinued treatment earlier, were not captured so the event rates found may not be a true reflection of events in the overall schizophrenia population. Secondly, retrospective chart review relies on a consistent and comprehensive clinical record, which may not always be the case, so clinical details may be missing for some patients. In addition, there may also have been misinterpretation of available data in the charts by reviewers or a lack of clarification of missing data. Thirdly, a power calculation was not completed *a priori* because the number of charts available was limited and the purpose of the review was exploratory. Finally, as discussed above, the small number of patients receiving RLAI beyond 18 months may have compromised the validity of estimates of treatment retention and hospitalization rates for this patient population. Nevertheless, despite these limitations, the study provides an assessment of usual care outcomes for schizophrenia patients receiving depot antipsychotic medications in a single Mental Health Centre in Canada.

## Conclusions

There were no significant differences between treatment retention and hospitalization rates in patients receiving RLAI or FGAI, although estimation of long-term rates for the former were based on small numbers of patients. However, RLAI treatment was associated with a significantly lower incidence of EPS, a trend towards a lower incidence of TD, and reduced utilization of oral antipsychotics. The results emphasize the need for a larger study comparing RLAI and FGAI treatment in schizophrenia, one powered to detect clinically relevant changes in patient outcomes.

## Abbreviations

RLAI: Risperidone long-acting injection; FGAI: First generation antipsychotic injections; EPS: Extrapyramidal symptoms; TD: Tardive dyskinesia; PMC: Partnership Medication Clinic; AIMS: Abnormal Involuntary Movement Scale; SAS: Simpson Angus Scale; KS: Kolmogorov-Smirnov; CI: Confidence interval; PANSS: Positive and negative Syndrome Scale.

## Competing interests

Dr. Richard Williams has received research grants and or honoraria from Janssen-Ortho Inc., Pfizer Canada Inc., AstraZeneca, Novartis, Eli Lily, Sanofi-aventis, Bristol Meyers Squibb, Organon Canada Inc., Solvay PharmaInc., and OBEcure Ltd. All other authors declare they have no conflicts of interest.

## Authors’ contributions

LL, BZ, and RW designed the study. LL completed all data collection, compiled the primary analysis, and wrote the first draft of the manuscript. BZ and RW provided clinical insight and expertise. All authors contributed to and have approved the final manuscript. All authors read and approved the final manuscript.

## Pre-publication history

The pre-publication history for this paper can be accessed here:

http://www.biomedcentral.com/1471-244X/13/155/prepub
